# Supplementary material for: Effects of Combinations of Untranslated-Region Sequences on Translation of mRNA
Source: Biomolecules. 2023 Nov 20;13(11):1677. doi: 10.3390/biom13111677 (PMC10669451; doi:10.3390/biom13111677)
Supplement: Supplementary file 1 [file biomolecules-13-01677-s001.zip › biomolecules-2728965-supplementary.pdf]

Supplement Table S1: 5′UTR and 3′UTR sequences

Name	UTR	Sequence
H4C2	5'UTR	GGGAAATAGTACCTGGTTTTTGTTGTCAGCTGGTTAGCCACC
HBB	5'UTR	GGGAAATACATTTGCTTCTGACACAACTGTGTTCACTAGCAACCTCAA ACAGCCACC
Rabb	5'UTR	GGGAAATACACTTGCTTTGACACAACTGTGTTTACTTGCAATCCCCCA AAACAGACAGACGCCACC
TPL	5'UTR	GGGAAATACTCTCTTCGCATCGCTGTCTGCGAGGGCCAGCTGTTGGGC TCGCGGTTGAGGACAAACTCTTCGCGGTCTTTCCAGTACTCTTGGATC GGAAACCCGTCGGCCTCCGAACGGTACTCCGCCACCGAGGGACCTGA GCGAGTCCGCATCGACCGGATCGGAAAACCTCTCGAGAAAGGCGTCT AACCAGTCACAGTCGCAAGAAGCTTAGCATTCCGGTAACTGTTGGTAA AGCCACC
RNA-1273 (Moderna)	5'UTR	GGGAAATAAGAGAGAAAAGAAGAGTAAGAAGAAATATAAGACCCCG GCGCCGCCACC
BNT162b2 (Bionthech)	5'UTR	GAGAATAAACTTGTATTCTTCTGGTCCCCACAGACTCAGAGAGAACCC GCCACC
mtRNR-EMCV	3'UTR	CAAGCACGCAGCAATGCAGCTCAAAACGCTTAGCCTAGCCACACCCC CACGGGAAACAGCAGTGATTAACCTTTGCAATAAACGAAAGTTTAAC TAAGCTATACTAACCCCAGGGTTGGTCAATTTCGTGCCAGCCACACCC TGGAGCTAGCGGGGCTGAAGGATGCCCAGAAGGTACCCCATTGTATG GGATCTGATCTGGGGCCTCGGTGCACATGCTTTACATGTGTTTAGTCG AGGTTAAAAAGCGTCTAGGCCCCCCGAACCACGGGGACGTGGTTTTCC TTTGAAAAACACGATGATAATATGGCCACAACCGGCTTCTGCCTAATA AAGAATGTTCAGCTCAA
mtRNR-mtRNR	3'UTR	CAAGCACGCAGCAATGCAGCTCAAAACGCTTAGCCTAGCCACACCCC CACGGGAAACAGCAGTGATTAACCTTTGCAATAAACGAAAGTTTAAC TAAGCTATACTAACCCCAGGGTTGGTCAATTTCGTGCCAGCCACACCC TGGAGCTAGCCAAGCACGCAGCAATGCAGCTCAAAACGCTTAGCCTA GCCACACCCCCACGGGAAACAGCAGTGATTAACCTTTGCAATAAACG AAAGTTTAACTAAGCTATACTAACCCCAGGGTTGGTCAATTTCGTGCC AGCCACACCCTGGAGCTAGC
mtRNR-AES	3'UTR	CAAGCACGCAGCAATGCAGCTCAAAACGCTTAGCCTAGCCACACCCC CACGGGAAACAGCAGTGATTAACCTTTGCAATAAACGAAAGTTTAAC TAAGCTATACTAACCCCAGGGTTGGTCAATTTCGTGCCAGCCACACCC TGGAGCTAGCCTCGAGCTGGTACTGCATGCACGCAATGCTAGCTGCCC CTTTCCCGTCCTGGGTACCCCGAGTCTCCCCCGACCTCGGGTCCCAGG TATGCTCCCACCTCCACCTGCCCCACTCACCACCTCTGCTAGTTCCAGA CACCTCC
RNA-1273 (Moderna)	3'UTR	GCTGGAGCCTCGGTGGCCTAGCTTCTTGCCCCTTGGGCCTCCCCCCAG CCCCTCCTCCCCTTCCTGCACCGTACCCCCGTGTCTTTGAATAAAGTCT GAGTGGGCGGCA
BNT162b2 (Bionthech)	3'UTR	CTCGAGCTGGTACTGCATGCACGCAATGCTAGCTGCCCCTTTCCCGTC CTGGGTACCCCGAGTCTCCCCCGACCTCGGGTCCCAGGTATGCTCCCA CCTCCACCTGCCCCACTCACCACCTCTGCTAGTTCCAGACACCTCCCA AGCACGCAGCAATGCAGCTCAAAACGCTTAGCCTAGCCACACCCCCA CGGGAAACAGCAGTGATTAACCTTTGCAATAAACGAAAGTTTAACTA AGCTATACTAACCCCAGGGTTGGTCAATTTCGTGCCAGCCACACCCTG GAGCTAGC
